# Hypoxia preconditioning protection of corneal stromal cells requires HIF1α but not VEGF

**Published:** 2009-05-18

**Authors:** Dongmei Xing, Joseph A. Bonanno

**Affiliations:** School of Optometry, Indiana University, Bloomington, IN

## Abstract

**Purpose:**

Hypoxia preconditioning protects corneal stromal cells from stress-induced death. This study determined whether the transcription factor HIF-1α (Hypoxia Inducible Factor) is responsible and whether this is promulgated by VEGF (Vascular Endothelial Growth Factor).

**Methods:**

Cultured bovine stromal cells were preconditioned with hypoxia in the presence of cadmium chloride, a chemical inhibitor of HIF-1α, and HIF-1α siRNA to test if HIF-1α activity is needed for hypoxia preconditioning protection from UV-irradiation induced cell death. TUNEL assay was used to detect cell apoptosis after UV-irradiation. RT-PCR and western blot were used to detect the presence of HIF-1α and VEGF in transcriptional and translational levels.

**Results:**

During hypoxia (0.5% O2), 5 μM cadmium chloride completely inhibited *HIF-1α* expression and reversed the protection by hypoxia preconditioning.  *HIF-1α* siRNA (15 nM) reduced *HIF-1α* expression by 90% and produced a complete loss of protection provided by hypoxia preconditioning.  Since VEGF is induced by hypoxia, can be HIF-1α dependent, and is often protective, we examined the changes in transcription of *VEGF* and its receptors after 4 h of hypoxia preconditioning.  *VEGF* and its receptors *Flt-1* and *Flk-1* are up-regulated after hypoxia preconditioning.  However, the transcription and translation of VEGF were paradoxically increased by siHIF-1α, suggesting that VEGF expression in stromal cells is not down-stream of HIF-1α.

**Conclusions:**

These findings demonstrate that hypoxia preconditioning protection in corneal stromal cells requires HIF-1α, but that VEGF is not a component of the protection.

## Introduction

Keratocyte apoptosis is the earliest stromal event noted after corneal epithelial injury and has an important role in the overall wound healing response [1]. Keratocyte loss promotes the activation and proliferation of surrounding keratocytes which leads to a change in gene expression and matrix production that can affect cornea clarity [2-5]. Preventing keratocyte loss has been suggested as a possible approach to reduce keratocyte activation and possible subsequent myofibroblast formation [6]. Hypoxia preconditioning has been shown to be protective in brain [7], bladder [8], and retina [9]. We have shown that hypoxia preconditioning provides generalized protection to corneal stromal cells against induced apoptosis in vitro and in an ex vivo cornea model. Cobalt chloride, which is a chemical inducer of HIF-1α, provided protection to corneal stromal cells in the absence of hypoxia [10]. The nuclear transcription factor HIF-1α (hypoxia inducible factor) is induced by hypoxia in these cells and protection is also provided by an HIF-1α inducer, Cobalt chloride (CoCl_2_), suggesting that HIF-1α is a necessary component of hypoxia preconditioning protection [10].

HIF-1α is the major transcription factor that controls the expression of hypoxia-regulated genes. To activate transcription of target genes, HIF-1α dimerizes with ARNT (aryl hydrocarbon receptor nuclear translocator) and binds to the HRE (hypoxia responsive element ). ARNT is constitutively expressed so the hypoxic induction and modification of HIF-1α determines the transcriptional activity. Under normoxic conditions, HIF-1α is continuously degraded in proteasomes. Oxygen-dependent hydroxylation of proline residues in the ODD domain of HIF-1α leads to interaction with the VHL (von Hippel Lindau) ubiquitin ligase complex. Furthermore, oxygen-dependent hydroxylation of asparagine in the CAD domain prevents interaction of HIF-1α with the p300/CBP coactivator that is needed to induce transcription [11]. HIF-1α levels are inversely related to oxygen tension with a half-maximal response at 1.5-2% O_2_ and a maximal response at 0.5% O_2_ [12]. HIF-1α has been shown to be pro-apoptotic and anti-apoptotic. Hypoxia increases the expression of Nips, a pro-apoptotic member of the Bcl-1 family in human tumor cells [13]. Hypoxia preconditioning can be anti-apoptotic either by HIF-1α dependent or independent pathways. For example, up-regulation of the anti-apoptotic protein IAP-2 by hypoxia does not require HIF-1α and is regulated by the NFκb pathway [14]. However, protection of cortical neurons [15,16], pancreatic cancer cells [16], and retinal photoreceptors require HIF-1α, which is generally associated with upregulation of protective growth factors such as VEGF (vascular endothelial growth factor) and EPO (erythropoietin).

The VEGF gene has HREs and is a well-known target gene regulated by HIF-1α. VEGF expression can be increased by hypoxia preconditioning [17] or over-expression of HIF [18]. VEGF has been shown to prevent vascular endothelial cell death down stream of HIF-1α by at least two previous studies [19,20]. VEGF, down-stream of HIF-1α, also protects cardiomyocytes following ischemia [21]. VEGF and other tyrosine kinase activated receptors activate PI-3K and akt (Protein Kinase B) leading to phosphorylation of apoptotic factors that ultimately suppress release of cytochrome C and activation of caspases [22]. A recent study however, has shown that VEGF expression can be HIF-1α independent as shown in skeletal muscle cells where VEGF is regulated by PGC-1α ( peroxisome proliferator activated receptor gamma coactivator-1 alpha ) [23].

In this study, we found that siRNA knockdown of HIF-1α abrogated hypoxia dependent protection of corneal stromal cells. Because VEGF production is increased during corneal hypoxia and VEGF has very strong protective functions in many systems, we examined VEGF expression during HIF-1α knockdown. We found that VEGF expression was actually increased indicating that it is not a component of hypoxia dependent cell protection.

## Methods

### Cell culture

Corneal stromal cells were cultured as previously described [10]. Briefly, blocks of stroma were cut from fresh bovine cornea and cultured in DMEM (GIBCO) supplemented with 10% fetal bovine serum (FBS), 100 units/ml of penicillin, 100 μg/ml of streptomycin, and 0.25 μg/ml of amphotericin B. Corneal stromal fibroblasts migrated from the stromal explants and grew exponentially at densities below 5×10^5^ cells/ml. Second to third generation fibroblasts were seeded onto coverslips or petri-dishes and used in all cell culture experiments.

### Induction of hypoxia

For hypoxia preconditioning, cells were placed in a hypoxia chamber (Coy Lab Products Inc., Grass Lake, MI) equilibrated with 5% CO_2_ and 0.5% oxygen-balance nitrogen for 4 h duration as indicated.

### UV-irradiation

Corneal fibroblasts (5×10^4^ cells) were sub-cultured to 25 mm coverslips in DMEM supplemented with 0.5% FBS for 2 days. Media was changed immediately before each experiment. This amount of serum was sufficient to prevent cell death, but does not promote proliferation. A germicidal lamp (TUV/30W/G30 T8; Philips Lighting Company, Somerset, NJ) that emitted radiation ranging from 230 to 400 nm was used as the UV source as previously described [10] Cells were irradiated for 2 min, which corresponds to 5.1 mJ/cm^2^. Cells were irradiated at 80-90% confluence. Culture media was removed and replaced with 2 ml of a balanced ringer’s solution to avoid variations in UV absorption from media components. After irradiation the ringer’s solution was discarded and replaced by fresh DMEM/0.5% FBS.

### TUNEL assay and cell counting

Four hours after UV-irradiation, cells on coverslips were fixed in 4% formaldehyde/PBS at 4 °C for 25 min. Following fixation the cells were rinsed twice with PBS and permeabilized with prechilled 0.2% Triton X-100/PBS on ice for 5 min. A fluorescence-based TUNEL assay was used according to the manufacturer's instructions (ApoAlert; BD Biosciences, Palo Alto, CA). Cells were counterstained with DAPI and mounted with prolong antifade reagent (Molecular Probes, Eugene, OR). Images were obtained with a fluorescence microscope (Nikon E600; Nikon, Melville, NY) equipped with a charge coupled device camera with active cooling system. For fibroblasts cultured on coverslips, five random distinct 200X microscopic fields were photographed on each coverslip. DAPI(+) cells were counted to obtain the total cell count. DAPI(+) and TUNEL(+) cells were counted as apoptotic cells. DAPI(−) and TUNEL(+) areas were considered artifacts and excluded from the count. Data was collected from about 750 cells for each condition in each experiment. Experiments were repeated at least three times giving a total of at least 2,000 cells counted per condition.

### RNA interference and cell transfection

Corneal fibroblasts (2×10^5^ cells) were sub-cultured to 60 mm petri-dishes or 5×10^4^ cells were sub-cultured to 25 mm coverslips in DMEM supplemented with 0.5% FBS for one day to reach 50% confluence. RNAi targeting HIF-1α was designed using Bos Taurus *HIF-1α* mRNA (GenBank NM_174339). The position for siRNA targeting starts at 1,450 of the *HIF-1α* mRNA. The sense 5’-AAG AAG GAG CCT GAT GCT TTA CCT GTC TC-3’ and antisense sequence 5’-AAT AAA GCA TCA GGC TCC TTC CCT GTC TC-3’, were synthesized and annealed as following the manufacturer’s protocol (Cat No. 1620; Ambion Inc., Austin, TX). Cells were transfected with the oligonucleotide duplexes for 6 h and then changed to regular medium. For mock transfection, cells were exposed to oligofectamine alone. A siRNA targeted to an irrelevant mRNA (Dharmacon Research, Chicago, IL) serves as non-targeting control.

### RT-PCR

Total RNA was isolated using TRIzol reagent (Invitrogen). cDNA synthesis was performed using Invitrogen Superscript III (200 U μl^−1^), Oligo dT_12–18_ primer and 1 μg mRNA as manufacture’s instructions. *VEGF*, *FLK-1*, and *Flt-1* primers were selected to amplify the 508 bp, 386 bp, and 334 bp fragments of mRNA respectively according to previous report [24]. Primer sequences: *VEGF* sense 5-TAC CTT CAC CAT GCA AG, *VEGF* antisense 5-CAC ATC TGC AAG TAC GTT CG; *FLK-1* sense 5’-TTC TTG CCC AAC AAT CAG AG, *FLK-1* antisense 5’-TAG CTG GGA ATA CTG AAG CC; *Flt-1* sense 5’-TAT AGC ACC AAG AGC GAC, *Flt-1* antisense 5’-GTG TCG AGT ACG TAA ACG. Each 25 ul of amplification reaction contains 0.4 μl Taq polymerase (cat. 92877933; Roche, Nutley, NJ), 2 μl of dNTP mix, 0.3 μm primers, 6 μl of cDNA for *Flt-1* and 2 μl of cDNA for everything else. The PCR parameters are 40 cycles as follow: denaturation at 94 °C for 15 s, annealing at 55–60 °C for 25 s, extension at 72 °C for 35 s, according to a previous report [24]. The PCR products were separated on 1.7% agarose electrophoresis gels and stained with 0.5 μg/ml ethidium bromide and recorded for analysis.

### Western blot analysis

Whole cell lysates were prepared as previously described [25]. Briefly, treated and untreated cells were extracted with lysis buffer (50 mmol/l Tris–HCl, pH 7.5, 5 mmol/l EDTA, 150 mmol/l NaCl, 0.5% Triton X-100, 10 mmol/l sodium fluoride, 20 mmol/l β-mercaptoethanol, 250 μmol/l sodium orthovanadate, 1 mmol/l PMSF, and complete protease inhibitor cocktail; Sigma, St Louis, MO), and incubated at 4 °C for 30 min. The lysates were sonicated and centrifuged at 14,000x g for 15 min. The supernatants were collected and stored at −80 °C. Protein concentrations were determined by the BCA method. Protein (50 μg) was separated on 8-12% polyacrylamide-SDS gel and electroblotted onto nitrocellulose membranes (Bio-Rad laboratories, Hercules, CA). After blocking with TBS/5% skim milk, the membrane was incubated overnight at 4 °C with primary antibodies against HIF-1α (Cat: MA1-516; ABR, Rockford, IL) at concentration of 1:2,000 or polyclonal antibody against VEGF (Cat: sc-507; Santa Cruz Biotechnology, Inc., Santa Cruz, CA) , at 1:200 followed by peroxidase conjugated anti-mouse IgG or anti-rabbit IgG for 1 hr at room temperature. Signals were detected with ECL. Data was analyzed using Un-scan-it gel analysis software (Silk Scientific, Orem, UT). Relative increase in protein expression compared to its own control is calculated.

### Statistical analysis

Data is presented as the mean±SE for at least three separate experiments. Student's *t*-test was employed for statistical analysis, with significant differences determined as p<0.05.

## Results

### Cadmium chloride prevents induction of HIF-1α and inhibits hypoxia preconditioning protection

Previously, we showed that 4 h of hypoxia preconditioning or application of CoCl_2_, a chemical HIF-1α inducer, provided protection against UV induced corneal stromal cell apoptosis [10], suggesting that HIF-1α has a role in preventing apoptosis. Conversely, treatment with low concentrations of cadmium chloride have been shown to inhibit the activation of HIF-1α by hypoxia [26-28]. Here we test whether cadmium chloride (5 μM) reduces HIF-1α activation by hypoxia in corneal stromal cells and whether it abrogates hypoxia preconditioning protection.

**Figure 1:**
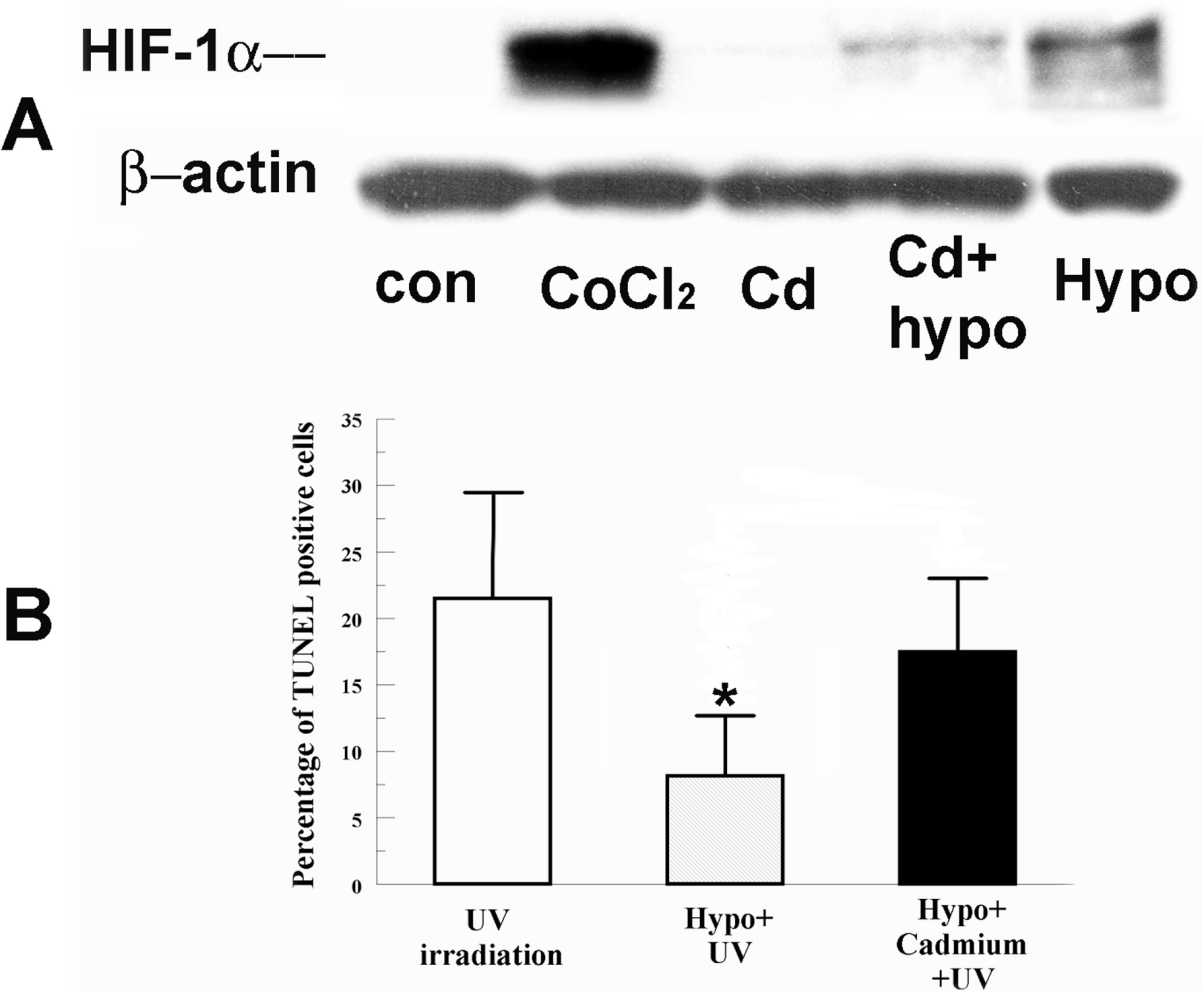
Effect of Cadmium on HIF-1α accumulation and hypoxia preconditioning protection. **A**: Cells were incubated under normoxia with 200 μM CoCl_2_ (as a positive control), 5 μM CdCl_2_ alone, hypoxia (0.5% O_2_) alone, or hypoxia with CdCl_2_ for 4 h. Whole cell lysates were collected immediately after treatment, separated on SDS-PAGE gel and blotted for HIF-1α. β-actin was detected as a loading control. **B**: Cells were pretreated with hypoxia with or without CdCl_2_ for 4 h and irradiated 2 min with UV. Four hours after irradiation, cells were fixed and stained for TUNEL. Bar graph shows percentage of TUNEL positive cells in indicated groups. Error bars represent standard error of the mean (n=3); the asterisk indicates significantly different from UV control (p<0.05).

Figure 1A shows that cadmium chloride significantly reduces the induction of HIF-1α by hypoxia. Figure 1B shows that hypoxia preconditioning significantly protected cells from UV irradiation. However, the addition of cadmium eliminated this protection. Cadmium alone had no significant effect on HIF-1α (figure 1A) or apoptosis (data not shown). These results show that decreased HIF-1α levels lead to reduction of protection, which suggests that HIF-1α is involved in the hypoxia protection.

### Characterization of RNAi targeting bovine *HIF-1α* in bovine corneal stromal cells

**Figure 2:**
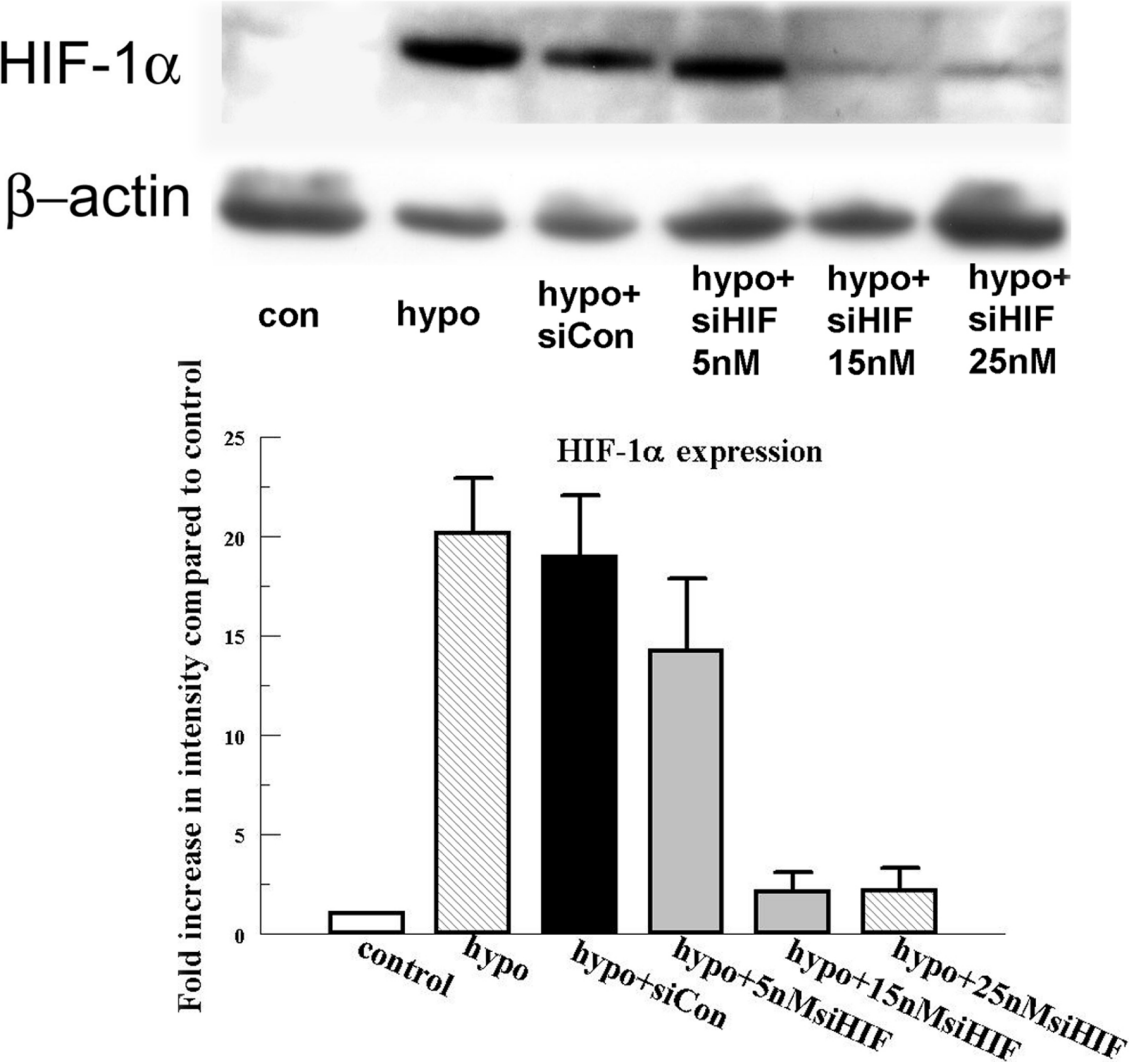
Effect of *HIF-1α* siRNA on HIF-1α protein expression. Cells were transfected with 15 and 25 nM *HIF-1α* siRNA and an siRNA non targeting control as indicated for 6 h. Twenty-four hours after transfection, cells were exposed to 4 h of hypoxia. Protein was collected immediately after treatment and blotted for HIF-1α. Bar graph represents the band intensity relative to the control. Error bars represent the standard error of the mean (n=3). β-actin is detected as loading control.

We designed a bovine specific *HIF-1α* small interference RNA. The efficiency and potency of this siRNA was tested in corneal stromal cells. Figure 2 shows that the *HIF-1α* siRNA produced a significant reduction in the hypoxia induced *HIF-1α* expression. The maximum effect (about 90% of reduction) of the siRNA could be achieved a concentration as low as 15 nM. Non targeting siRNA control did not significantly affect the HIF-1α level.

### Complete loss of hypoxia preconditioning protection by siHIF-1α

**Figure 3:**
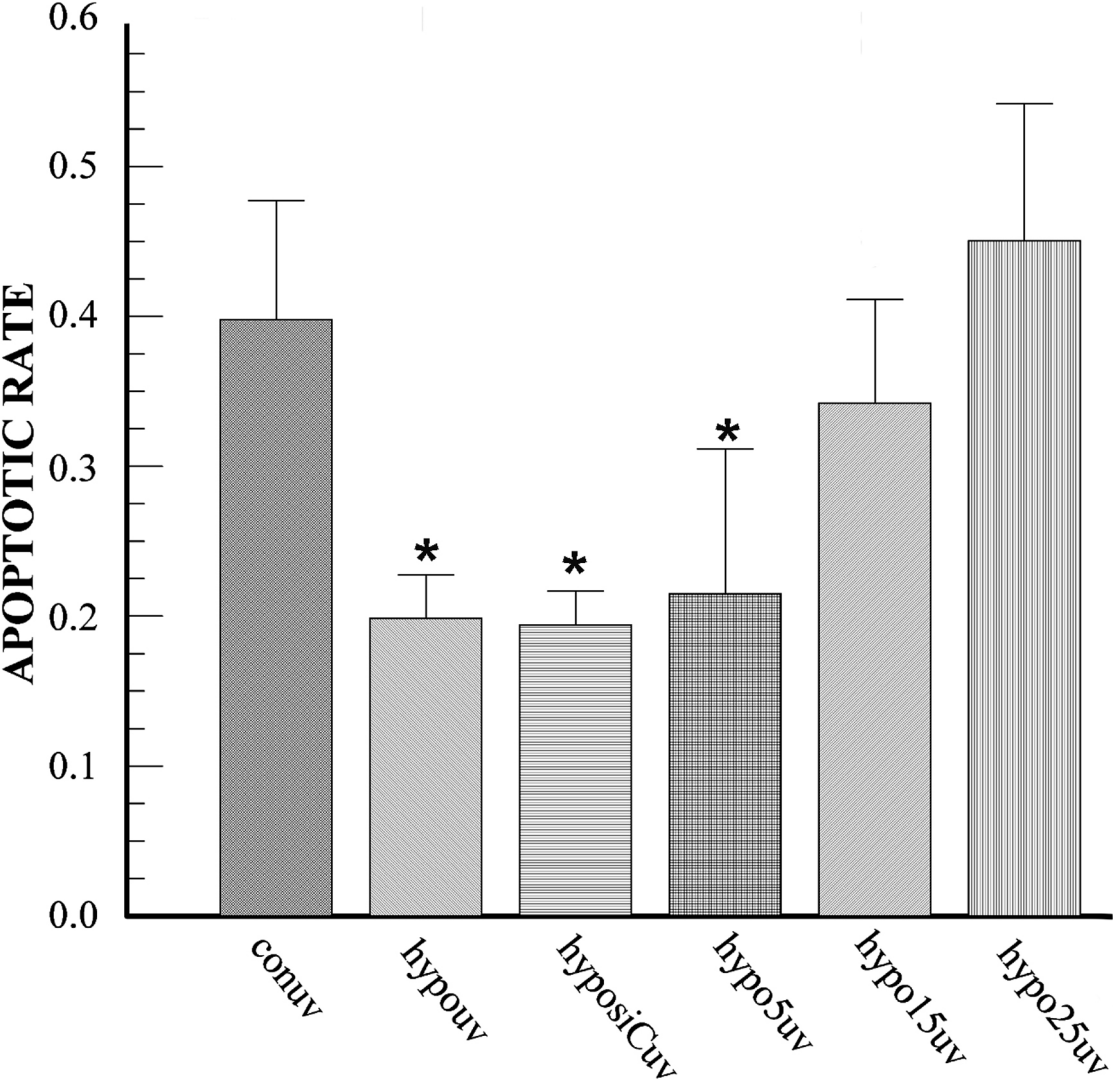
Effect of *HIF-1α* siRNA on hypoxia preconditioning protection. Bovine stromal cells were transfected with the 15 nM (hypo15uv) and 25 nM (hypo25uv) *HIF-1α* siRNA and non targeting siRNA control (labeled as hyposiCuv) for 6 h. Twenty-four hours later, cells were exposed to hypoxia for 4 h and then stressed with UV-irradiation for 2 min. Cells were stained with TUNEL 4 h after irradiation. Error bars represent the standard error of the mean (n=3). The asterisk indicates statistically different from control UV irradiation.

To definitively determine that *HIF-1α* is involved in hypoxia protection, we tested the effect of *HIF-1α* siRNA on hypoxia protection against UV-irradiation induced apoptosis in corneal stromal cells. Figure 3 shows that UV irradiation induced a 40±8.5% apoptotic rate whereas hypoxia preconditioning reduces this apoptotic rate to 20±3.0%. This is a similar protective effect to that reported previously [10]. Fifteen and 25nM siRNA targeting *HIF-1α* eliminates the hypoxia preconditioning protection, bringing the apoptotic rate back to 37.5±5.5% and 42±10.1%, respectively. These results demonstrate that hypoxia protection requires *HIF-1α* expression.

### *VEGF* is induced by hypoxia preconditioning in bovine corneal stromal cells

**Figure 4:**
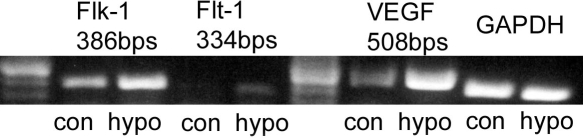
Up-regulation of *VEGF* and its receptors by hypoxia in bovine keratocytes. Bovine stromal cells were treated with hypoxia for 4 h. Total RNA was collected immediately after treatment. Image shows RT-PCR analysis for *VEGF*, *FLK-1*, and *Flt-1*. *GAPDH* was detected as an internal control. Representative image of three experiments is shown.

Genes prominently induced by hypoxia include growth factors like *VEGF* and *EPO* [29,30]. Among these growth factors, *VEGF* was found to be up-regulated in most cell types [31]. We tested here whether the transcription of *VEGF* and its receptors *Flk-1* and *Flt-1* are up-regulated in corneal stromal cells by hypoxia preconditioning. Figure 4 shows that *VEGF* is prominently up-regulated by hypoxia and both *VEGF* receptors *Flk-1* and *Flt-1* are also up-regulated. The transcription of receptor *Flt-1* is significantly lower than *Flk-1* in corneal stromal cells.

### Induction of *VEGF* by hypoxia preconditioning is not reduced by siHIF-1α

**Figure 5:**
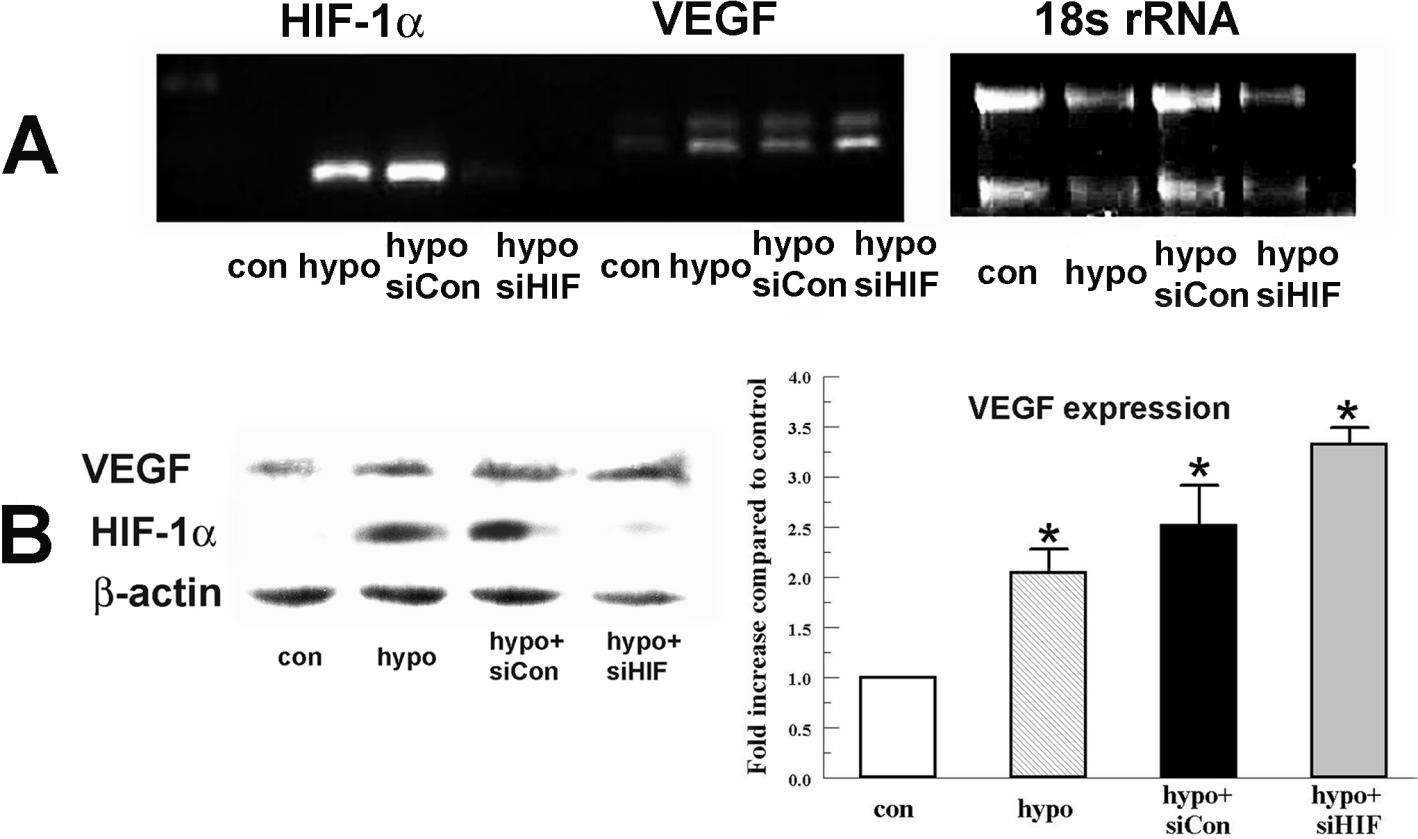
Effect of *HIF-1α* siRNA on hypoxia-induced *VEGF* expression. Bovine stromal cells were transfected with *HIF-1α* siRNA or non-targeting siRNA control for 6 h. Twenty-four hours after transfection cells were preconditioned with hypoxia for 4 h. **A**: Total RNA was collected immediately after treatment. Image shows RT-PCR analysis for *HIF-1α* and *VEGF*. 18s rRNA was detected as an internal control. **B**: Protein analysis of VEGF and HIF-1α. Whole cell lysates were collected immediately after treatment, separated by SDS PAGE and probed for VEGF and HIF-1α. β-actin was detected as a loading control. Bar graph shows VEGF expression in indicated groups relative to control. Error bars represent standard error of the mean (n=3).

*VEGF* has been shown to be directly up-regulated by HIF-1α in vascular endothelial cells and kidney [32,33]. But recent evidence in skeletal muscle showed that *VEGF* expression is completely independent of HIF-1α [23]. We tested whether *VEGF* is down stream of HIF-1α in bovine corneal stromal cells. Figure 5A shows that *VEGF* mRNA is increased after hypoxia treatment but it is not reduced by *HIF-1α* siRNA. Figure 5B shows that *HIF-1α* siRNA significantly reduces the HIF-1α level, but it does not reduce *VEGF* expression. On the contrary, *VEGF* expression under hypoxia treatment with *HIF-1α* siRNA increases 3.3±0.1 fold compared to control which is significantly higher compared to hypoxia alone (2.1±0.3 fold increase).

## Discussion

The concept of hypoxia preconditioning protection is well documented in many tissues and various factors are demonstrated to participate in the protection [9,34-37]. The production of EPO during whole body hypoxia protected photoreceptors from light induced cell death [9]. Hypoxia is also known to stimulate translocation of hsp27 and αB-crystallin from diffuse locations to defined structures, which is associated with a decrease in caspase-3 activity [38,39]. HIF-1α is a major modulator in the hypoxic environment. It is generally considered to play a protective role and induces the up-regulation of other protective factors like Hsp27 [40] and VEGF [32].

VEGF has been shown to be protective in several cell types such as skeletal muscle and kidney cells [23,32]. But whether the effect of VEGF on protection is dependent on HIF-1α is cell type specific. In this study, we used cadmium chloride to reduce the induction of HIF-1α. Cadmium chloride 5 μM, in the presence of hypoxia, completely blocks HIF-1α induction by hypoxia (Figure 1A). UV induced apoptosis of cells preconditioned by hypoxia in the presence of cadmium was not significantly different from UV alone, suggesting that prevention of HIF-1α induction is necessary for protection.

The *HIF-1α* specific siRNA efficiently reduces HIF-1α expression by 90% at 15 nM (Figure 2B). This reduction in HIF-1α completely eliminates the protection provided by hypoxia preconditioning (Figure 3) indicating that HIF-1α is essential for this protection. A similar conclusion has been drawn from a mouse study where complete loss of hypoxia protection was due to partial deficiency of HIF-1α [41].

The regulation of *VEGF* in response to hypoxia can be mediated by HIF-1α in a number of tissues [32,42]. Our result indicates that *VEGF* expression is up-regulated by hypoxia (figure 4), but that this increase in *VEGF* transcription and expression is independent of HIF-1α (figure 5). Therefore, protection by hypoxia depends on HIF-1α, but not VEGF in corneal stromal cells. A recent study on rat heart also showed that HIF-1α is protective, but is not VEGF or EPO dependent [43]. The regulation of *VEGF* expression must be cell type specific since in kidney [32] and vascular endothelial cells [33] VEGF, which is protective, is dependent on HIF-1α. On the other hand, *VEGF* expression is totally HIF independent in skeletal muscle cells where *VEGF* is regulated by peroxisome proliferator activated receptor gamma coactivator-1 alpha (PGC-1α) [23]. Interestingly, a recent study has shown that *VEGF* expression is regulated by HIF-2α in human lung endothelial cells [44]. Further studies are needed to determine the mechanism for *VEGF* regulation in corneal stromal cells.

Overall, the results from this study show that hypoxia preconditioning protection requires induction of *HIF-1α*. A likely protective factor, VEGF, is up-regulated by hypoxia preconditioning, but is not induced by HIF-1α, indicating that VEGF is not the protective factor during hypoxia preconditioning. We have preliminary evidence to suggest that the PI-3K and akt pathways are activated by hypoxia. This suggests that Receptor Tyrosine Kinase ligands other than VEGF, (e.g., EPO) are required for protection in corneal stromal cells. Further studies are needed to determine these factors induced by HIF-1α that may protect corneal stromal cells during hypoxia preconditioning.
